# Homeostatic role of heterosynaptic plasticity: models and experiments

**DOI:** 10.3389/fncom.2015.00089

**Published:** 2015-07-13

**Authors:** Marina Chistiakova, Nicholas M. Bannon, Jen-Yung Chen, Maxim Bazhenov, Maxim Volgushev

**Affiliations:** ^1^Department of Psychology, University of ConnecticutStorrs, CT, USA; ^2^Department of Cell Biology and Neuroscience, University of California, RiversideRiverside, CA, USA

**Keywords:** synaptic plasticity, homosynaptic plasticity, STDP, Hebbian plasticity, homeostasis, heterosynaptic plasticity, runaway dynamics

## Abstract

Homosynaptic Hebbian-type plasticity provides a cellular mechanism of learning and refinement of connectivity during development in a variety of biological systems. In this review we argue that a complimentary form of plasticity—heterosynaptic plasticity—represents a necessary cellular component for homeostatic regulation of synaptic weights and neuronal activity. The required properties of a homeostatic mechanism which acutely constrains the runaway dynamics imposed by Hebbian associative plasticity have been well-articulated by theoretical and modeling studies. Such mechanism(s) should robustly support the stability of operation of neuronal networks and synaptic competition, include changes at non-active synapses, and operate on a similar time scale to Hebbian-type plasticity. The experimentally observed properties of heterosynaptic plasticity have introduced it as a strong candidate to fulfill this homeostatic role. Subsequent modeling studies which incorporate heterosynaptic plasticity into model neurons with Hebbian synapses (utilizing an STDP learning rule) have confirmed its ability to robustly provide stability and competition. In contrast, properties of homeostatic synaptic scaling, which is triggered by extreme and long lasting (hours and days) changes of neuronal activity, do not fit two crucial requirements for a hypothetical homeostatic mechanism needed to provide stability of operation in the face of on-going synaptic changes driven by Hebbian-type learning rules. Both the trigger and the time scale of homeostatic synaptic scaling are fundamentally different from those of the Hebbian-type plasticity. We conclude that heterosynaptic plasticity, which is triggered by the same episodes of strong postsynaptic activity and operates on the same time scale as Hebbian-type associative plasticity, is ideally suited to serve a homeostatic role during on-going synaptic plasticity.

## Introduction: three forms of synaptic plasticity

Normal operation of the brain requires the maintenance of balances across various neuronal and synaptic features, and keeping key factors within their operating ranges. This is achieved by a variety of homeostatic mechanisms operating at multiple nested levels, such as maintenance of excitation/inhibition balance and total activity at the network level, or homeostasis of synaptic weights at the single-cell level (van Vreeswijk and Sompolinsky, 1996; Haydon, 2001; Burrone and Murthy, 2003; Turrigiano, 2011; Davis, 2013). Synaptic weights are subject to changes caused by a variety of mechanisms mediating multiple forms of plasticity (Bliss and Collingridge, 1993; Abbott and Nelson, 2000; Malenka and Bear, 2004; Sjostrom et al., 2007; Chistiakova and Volgushev, 2009; Feldman, 2009). In the present review we will consider the relation between different forms of plasticity and synaptic homeostasis. We ask: which forms of plasticity bring synaptic weights out of balance and which forms may serve to restore their balance?

**Figure 1:**
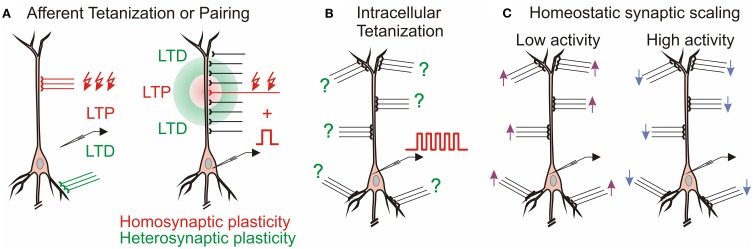
**Multiple forms of plasticity at cortical synapses. (A)** Homosynaptic and heterosynaptic plasticity. Left: In CA1 region of the hippocampus, LTP at Schaffer collateral inputs (red) induced by afferent tetanization is accompanied by heterosynaptic LTD at commissural inputs to basal dendrites (Lynch et al., 1977). Right: Mexican-hat profile of plasticity in hippocampus (White et al., 1990) and amygdala (Royer and Paré, 2003). **(B)** Heterosynaptic plasticity can be induced by purely postsynaptic protocol, intracellular tetanization—bursts of spikes evoked by depolarizing pulses without presynaptic stimulation in hippocampus (Kuhnt et al., 1994) and neocortex (Volgushev et al., 1994, 1997). Question marks denote that individual synapses may undergo LTP or LTD or do not change after the same induction. **(C)** Homeostatic synaptic scaling: prolonged (hours/days) changes of activity lead to compensatory up-scaling or down-scaling of synaptic weights (Turrigiano et al., 1998).

The multitude of forms of synaptic plasticity can be segregated into three broad types, distinguished by the differential activity patterns required for their induction, distinct functions served in learning systems, and diverse computational roles. The first, *homosynaptic plasticity*, requires presynaptic activation of the synapse for the induction. By definition, it occurs only at synapses that were directly involved in activation of a cell during the induction, for example during afferent tetanization or pairing procedure (Figure 1A). This form of plasticity is also called input specific, and, if the induction follows Hebbian-type learning rules, associative (Bliss and Collingridge, 1993). For induction of associative plasticity correlated activity of pre and postsynaptic neurons is crucial. Associative plasticity underlies a multitude of phenomena in the nervous system, ranging from refinement of connectivity during development (“neurons that fire together wire together”) to extraction of causal relations between events in the environment in Pavlovian conditioning and other types of associative learning as well as motor learning. The second form, *heterosynaptic plasticity*, can be induced at synapses that were not active during the induction of homosynaptic plasticity. Thus, it is not limited to active synapses, but may also change non-active synapses after episodes of strong postsynaptic activity. As the majority of synapses onto a cell are not presynaptically activated during a typical induction protocol, heterosynaptic plasticity typically affects a larger population of synapses than homosynaptic plasticity does. Heterosynaptic plasticity can be induced at unstimulated synapses by typical induction protocols, such as afferent tetanization or a pairing procedure (Figure 1A), but also by purely postsynaptic protocols such as intracellular tetanization: bursts of spikes evoked by depolarizing pulses (Figure 1B). Homosynaptic and heterosynaptic plasticity have complementary computational properties, making both forms necessary for normal operation of neural systems with plastic synapses (Chistiakova and Volgushev, 2009; Chen et al., 2013; Chistiakova et al., 2014).

The third form of plasticity considered in this review is *homeostatic synaptic scaling*, which is induced by prolonged (hours/days) and dramatic changes of activity, and leads to compensatory scaling of synaptic weights (Turrigiano et al., 1998, recently reviewed in Burrone and Murthy, 2003; Turrigiano and Nelson, 2004; Rich and Wenner, 2007; Rabinowitch and Segev, 2008; Watt and Desai, 2010; Turrigiano, 2012). Prolonged silencing of neurons by tetrodotoxin (TTX) leads to up-scaling of synaptic weights, while prolonged elevation of activity leads to down-scaling of the synapses (Figure 1C). Homeostatic synaptic scaling is triggered by deviation of firing rate from a target level (Turrigiano et al., 1998; van Rossum et al., 2000) and can be considered as a mechanism of firing rate homeostasis. Computationally, homeostatic synaptic scaling may have the effect of a “delayed” normalization if changes of postsynaptic firing were due to changes at a subset of inputs, or scale all synapses proportionally if postsynaptic firing rates were changed because of uniform silencing (or uniform up-regulation of activity) at all inputs. Because homeostatic synaptic scaling is triggered by overall activity level, irrespective of which specific synapses contributed to the activation of a neuron, but changes the weights of all synapses of a cell proportionally, it may include changes of both homosynaptic (those which were active) and heterosynaptic (those which were not active) inputs.

In this review we will consider: (i) Why do learning systems need forms of plasticity, additional to homosynaptic plastic changes governed by associative, Hebbian-type learning rules? (ii) What are the requirements for these additional forms of plasticity, outlined in theoretical and modeling studies? (iii) Which biological candidate mechanisms express properties that fulfill these requirements?

## Homosynaptic plasticity: why it cannot work alone

Synaptic plasticity induced according to Hebbian-type rules mediates the formation and refinement of connectivity patterns in the nervous system during development, and underlies various types of associative learning throughout life. However, for at least two reasons, associative synaptic plasticity governed by Hebbian-type rules cannot be the only type of plasticity in a learning system. First, Hebbian-type learning rules impose an intrinsic positive feedback on synaptic weight changes, thus making the system prone to runaway dynamics of synaptic weights and activity. Second, they introduce only a weak degree of competition between synapses. Competition is indispensable for building sensory representations during development, and is instrumental in learning that involves differentiation.

**Figure 2:**
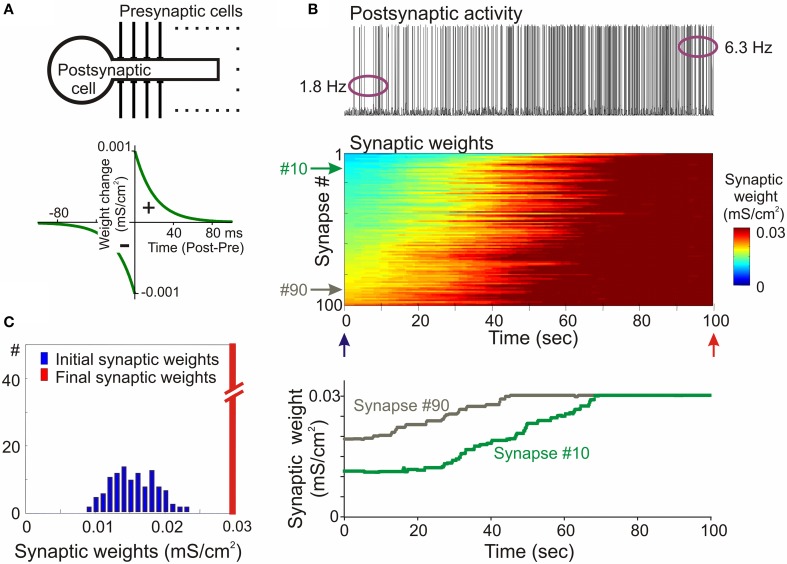
**Synaptic activity produced by weakly correlated inputs leads to runaway dynamics of synaptic weights in a model with symmetrical STDP mechanism. (A)** A scheme of a model neuron and STDP learning rule. The model neuron consisted of axosomatic and dendritic compartments, receiving 100 synaptic inputs from 100 presynaptic neurons. Each presynaptic neuron fired action potentials at ~1 Hz, with Poisson distributed interspike intervals. In simulations shown in this figure, firing of input neurons was mildly correlated (averaged cross-correlation 0.348 + −0.05). The STDP learning rule had symmetrical potentiation and depression windows (τ^+^ = τ^−^ = 20 ms; a^+^ = a^−^ = 10^−3^ mS/cm^2^). **(B)** Membrane potential trace of the model neuron (top); changes of the weights of 100 synapses (middle), color coded, with synapses sorted by their synaptic weights at the beginning of experiment; and changes of the weights of synapses #10 and #90 (bottom). In this model with symmetrical STDP learning rule synaptic inputs expressed runaway dynamics, and all inputs were potentiated to the maximum by the end of the simulation. **(C)** Distributions of synaptic weights at the beginning (blue, at 20 ms) and at the end (red, at 100 s) of simulation experiment shown in **(B)**. Note runaway dynamics of synaptic weights leading to their saturation at the extreme value (0.03 mS/cm^2^; red bar length is out of scale) and associated increase of the firing rate of postsynaptic neuron from 1.8 to 6.3 Hz. (Modified, with permission from Chen et al., 2013).

**Figure 3:**
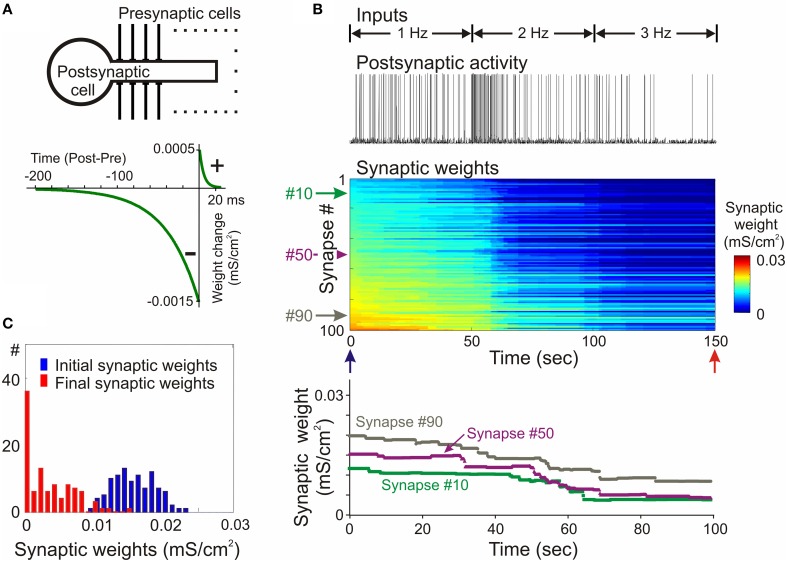
**Synaptic activity produced by weakly correlated inputs leads to runaway dynamics of synaptic weights in a model with negatively biased STDP mechanism. (A)** A scheme of the model neuron and STDP learning rule. The STDP learning rule with negative bias (τ^+^ = 5 ms, a^+^ = 0. 5 × 10^−3^ mS/cm^2^, τ^−^ = 40 ms, a^−^ = 1.5 × 10^−3^ mS/cm^2^). **(B)** Membrane potential trace of the model neuron (top) receiving input from presynaptic neurons firing at an average rate of 1 Hz during first 50 s of simulation, 2 Hz during 50–100 s and 3 Hz during 100–150 s, as indicated; changes of the weights of 100 synapses (middle), color coded, with synapses sorted by their synaptic weights at the beginning of experiment; and changes of the weights of synapses #10, #50, and #90 (bottom). In this model with negatively biased STDP learning rule synaptic inputs expressed runaway dynamics toward the minimum value. **(C)** Distributions of synaptic weights at the beginning (blue, at 20 ms) and at the end (red, at 150 s) of simulation experiment shown in **(B)**. Note runaway dynamics of synaptic weights leading to saturation at zero of about 40% of synapses, and associated dramatic decrease of postsynaptic firing rate despite a 3-fold increase of presynaptic firing. (Modified, with permission from Chen et al., 2013).

The propensity for runaway dynamics in a system with Hebbian-type synapses originates from the positive feedback on synaptic weight changes intrinsic to this type of plasticity rule. Potentiation, by making synapses stronger, increases the chance that these synapses will contribute to the firing of a neuron, and will be potentiated further. Similarly, depression of synapses decreases the chance that these synapses will contribute to the firing of a neuron, and thus decreases their chances for subsequent potentiation, but increases the probability that they undergo further depression. Indeed, neuron models with Hebbian-type rules for synaptic plasticity expresses runaway dynamics of synaptic weights. In a computational model of a single neuron with symmetrical windows for potentiation and depression within STDP rules, and receiving inputs from neurons with Poisson-distributed spikes intervals, synaptic weights get potentiated to the maximal value (Figure 2). In the same model but with STDP rules biased toward depression, synapses tend to be depressed, with the weights of many of them declining to zero (Figure 3). As a result, with an unchanged level of presynaptic activity the runaway of synaptic weights causes runaway dynamics of postsynaptic spiking. Runaway potentiation of synaptic weights to maximal values leads to overexcitability of neurons and excessive postsynaptic activity. In the Figure 2 model, firing rate of the postsynaptic model neuron increased from 1.8 Hz during the first 10 s, to 6.3 Hz during the last 10 s of simulation. Runaway depression leads to a decrease of postsynaptic activity, and eventually to the silencing of neurons. In the example in Figure 3, the postsynaptic neuron becomes essentially silent by the end of simulation despite a three-fold increase of the firing rate of presynaptic neurons. Both runaway potentiation and runaway depression scenarios lead to the disturbance of input-output relations of neurons (Chen et al., 2013), thus compromising computational abilities of neuronal networks (Skorheim et al., 2014). Moreover, runaway potentiation of synaptic weights and associated runaway activity are energetically unsustainable and may lead to pathology. To counteract runaway dynamics of synaptic weights and activity imposed by Hebbian-type plasticity rules, additional plasticity mechanisms are necessary. Such mechanisms would keep synaptic weights and neuronal networks with plastic synapses within their operational range, and thus maintain homeostasis of synaptic weights and neuronal activity despite unbalancing perturbations introduced by associative plasticity.

The idea of competition, which maintains that while undergoing changes, synapses might compete for shared limited resources such as available energy, molecules or plasticity factors, is well-grounded from the biological perspective (e.g., Miller, 1996; Peters et al., 2004). Synaptic competition is absolutely necessary for the development of sensory representations (Wiesel and Hubel, 1963; Aitkin et al., 1970; Merzenich et al., 1975; Thompson et al., 1983; Feldman, 2009), and is instrumental in a broad class of learning tasks that involve discrimination (Skorheim et al., 2014). However, Hebbian-type learning rules introduce only a weak, if any, degree of competition (Miller, 1996), restricted to the synapses receiving distinct input patterns (Zhang et al., 2011; see below for further discussion). Therefore, to support intrinsic competition between synapses, a mechanism for plastic changes outside of the Hebbian learning rule is required.

To summarize, homosynaptic plasticity, while being a major driving force for synaptic changes mediating associative learning, imposes positive feedback on synaptic changes which creates energetically and computationally unstable runaway dynamics. It also does not provide the required degree of synaptic competition known to be necessary for many types of learning. Modeling studies show the need for additional mechanisms which could constrain and balance Hebbian plasticity. Biological neuronal systems do possess such mechanisms, as evidenced by stable operation and diverse dynamics of synaptic changes over a broad range of conditions. It has not been clear, however, which of the multitude of proposed physiological mechanisms is/are able to serve these roles.

## Modeling perspective

### The need for homeostasis

The need for maintaining homeostasis of key variables of neuronal operation, such as synaptic weights, the amount of synaptic drive received by a neuron and neuronal activity, as well as for strong synaptic competition in learning systems with Hebbian-type rules has been appreciated since early theoretical studies (von der Malsburg, 1973; Miller and MacKay, 1994; Miller, 1996). To some extent, this can be achieved by careful adjustment of STDP rules. With an appropriate negative bias between the windows for potentiation and depression, STDP can indeed lead to stabilization of the neurons' mean firing rate (Song et al., 2000; Kempter et al., 2001; Gütig et al., 2003; Babadi and Abbott, 2010). For certain ranges of presynaptic firing rates, fine-tuned STDP can also support synaptic competition, by driving synaptic weights to the maximal value or to zero (e.g., Song et al., 2000; van Rossum et al., 2000; Gütig et al., 2003; Morrison et al., 2008). In all of these scenarios, the steady state distribution of synaptic weights depended on the fine-tuning of model parameters, such as the firing rate, the overall balance between excitatory and inhibitory inputs, and the fine details of plasticity rules (e.g., temporal shift or temporal jitter of plasticity windows, or weight-dependence of synaptic changes Song et al., 2000; Gütig et al., 2003; Morrison et al., 2008; Babadi and Abbott, 2010).

A problem with this solution is that it works only for certain combinations of internal features (STDP rules) and external events, input activity pattern and correlations. For STDP rules, it imposes strict requirements on the relative strength (amplitude and duration) of potentiation and depression windows which are compatible with the stable operation of a neuron (see below, **Figure 7A** and related text). Experimental evidence, however, has demonstrated an enormous heterogeneity in the width and magnitude of STDP windows in different synapses, cells, developmental stages, and conditions of neuromodulation (Nishiyama et al., 2000; Sjöström et al., 2001; Zhou et al., 2005; Haas et al., 2006; Feldman, 2009). Although depression-biased STDP rules had been reported for some synapses, a general requirement for a negative integral of STDP rules is not compatible with the experimentally observed variety of learning rules. Moreover, STDP rules adjusted to maintain stability in a neuron subject to a certain pattern of external drive may still lead to runaway dynamics when activity changes, for example if the level of correlation of activity at a subset of the inputs were increased (Gütig et al., 2003).

Positive feedback on synaptic changes can be counteracted by weight-dependence of plastic changes—a mechanism suggested theoretically (Oja, 1982) and confirmed experimentally (Bi and Poo, 1998; van Rossum et al., 2000; Hardingham et al., 2007). Weight dependence of plastic changes dictates that weaker synapses can potentiate more, while stronger synapses express less potentiation, and in the limit do not change or even depress (Hardingham et al., 2007). Depending on the details of implementation, weight dependence imposes bounds on synaptic weights without preventing their saturation at extreme values or grouping around the values over which an amplitude increase turns into a decrease. Results of theoretical analyses and computer simulations show that stability of the activity level of model neurons can indeed be achieved by implementing weight-dependence of plastic changes (van Rossum et al., 2000; Gütig et al., 2003). Weight-dependence is often used in combination with depression–biased STDP rules, which further improves the stability of the system (Kempter et al., 2001; Gütig et al., 2003; Morrison et al., 2008; Gilson and Fukai, 2011).

### Normalization as mechanism of stability

A simple and robust method of stabilization of the synaptic drive of neurons and of neuronal activity is normalization. Normalization has been commonly used in modeling and theoretical analyses of neurons and networks with plastic synapses since early studies (von der Malsburg, 1973; Oja, 1982). The concept of normalization is that following an induction of plasticity in a subset of synapses on a cell, the weights of all synapses on that cell are readjusted so that their sum (or squared sum) remains constant. Normalization has typically been implemented via multiplicative or subtractive methods. In multiplicative normalization (von der Malsburg, 1973; Oja, 1982), each weight is multiplied by an amount necessary to maintain the overall net weight (van Ooyen, 2001). In the subtractive method, all synaptic weights are changed by a fixed amount regardless of their weight: a decrease to compensate for the effect of homosynaptic potentiation or an increase to compensate for the effect of homosynaptic depression (Miller and MacKay, 1994). Normalization, either multiplicative or subtractive, robustly prevents runaway dynamics of activity, because it maintains synaptic drive of a cell at a certain level. It does not, however, prevent runaway dynamics of individual synaptic weights. Indeed training of models with normalization typically leads to a bimodal distribution of synaptic weights, with the weights of “winner” synapses bunched around the maximal value and weights of other synapses gathered around zero (e.g., Song et al., 2000; van Rossum et al., 2000; Gütig et al., 2003; Morrison et al., 2008).

Both methods of normalization introduce synaptic competition (see below). The use of normalization for achieving both stability of the activity level and synaptic competition has been further elaborated in later studies (e.g., Kempter et al., 2001; Elliott and Shadbolt, 2002; Wu and Yamaguchi, 2006; Finelli et al., 2008).

Note that because normalization affects all synapses of the neuron irrespective of their recent activity, it postulates the existence of heterosynaptic plasticity—changes of synapses which were not activated during plasticity induction.

### Homeostatic mechanism: the trigger and the timescale

In most modeling studies homeostasis of total synaptic weight and activity is achieved by normalization, implemented directly into the learning rules which update synaptic weights in each iteration. This introduces to the models, as an automatic consequence of their design, two important features: first, the trigger for plasticity also inevitably triggers the normalization, and second, the normalization operates on the same time scale as the plasticity rule. These two features may represent additional characteristics required from a homeostatic mechanism in systems with Hebbian-type learning rules.

The trigger for the homeostatic mechanism which protects synaptic weights and neuronal activity from runaway in the face of Hebbian plasticity is not unequivocally identified. The operation of neurons and neuronal networks is regulated by a multitude of homeostatic mechanisms operating at different levels, with a number of parameters that are tracked and a number of signals that could trigger homeostatic mechanisms. At the single-neuron level, biologically plausible candidates that regulate plasticity and constrain total synaptic weights could range from changes of the level of activity-dependent calcium influx (e.g., Yeung et al., 2004), to competition for limited available energy, intracellular resources or neurotrophic factors (e.g., Frey and Morris, 1997, 1998; Elliott and Shadbolt, 2002; Fonseca et al., 2004). These processes are initiated by the same signals which trigger synaptic plasticity (e.g., rise of intracellular calcium), and therefore by design accompany synaptic plasticity and operate on a similar time scale. At the network level, changes of the firing rate of a neuron or a neuronal population can be used as a trigger for homeostatic synaptic scaling (e.g., van Rossum et al., 2000; Zenke et al., 2013). These possibilities are not mutually exclusive, and some are inter-related, e.g., changes of firing rate inevitably lead to changes of calcium influx in the dendrites via voltage-gated calcium channels activated by backpropagating spikes (Spruston et al., 1995; Golding et al., 2002; Waters et al., 2003; Sjöström and Häusser, 2006). Because of their relation to global variables such as concentration of calcium in neurons or firing rate of neuronal populations, mechanisms of homeostatic regulation of synaptic weights might be embedded into a multi-level system of neuronal homeostasis and thus could be triggered by signals from several different levels.

The time scale on which mechanism(s) of synaptic homeostasis should operate is better understood. Results from the studies in which normalization was implemented as a separate mechanism with an individual time constant converge at the conclusion that to effectively counteract runaway dynamics imposed by associative plasticity this time constant should be short. In one of the first models which implemented homeostatic scaling of synaptic weights regulated by changes of the postsynaptic firing rate, a relatively short time constant of 100 s was used (van Rossum et al., 2000). This time scale is comparable to the time scale of synaptic changes induced by STDP or other Hebbian-type rules. Although in the model of van Rossum et al. (2000) the homeostatic scaling was primarily used to introduce synaptic competition, the same mechanism by design stabilizes the activity level. In a theoretical analysis of one-trial sequence learning of place-fields in the hippocampus, Wu and Yamaguchi (2006) concluded that for learning processes that occur within minutes, the physiological mechanism that constrains synaptic weights must also operate rapidly. The relation between the timescale of a homeostatic mechanism and its ability to maintain the stability of a system with Hebbian-type plastic synapses has been directly addressed in a recent study by Zenke et al. (2013). The authors found that to achieve robust stability of the system, Hebbian-type plastic synapses must be complemented by a homeostatic mechanism operating on a time scale of seconds to minutes, which is comparable to the time scale of plasticity itself (Zenke et al., 2013). This conclusion is compatible with a wealth of evidence from computational studies. In fact, to the best of our knowledge, in all computer models which used normalization or mechanisms inspired by homeostatic synaptic scaling for the purpose of preventing runaway dynamics and/or introducing synaptic competition, these mechanisms were implemented on a fast time scale, the same or comparable to the time scale of the Hebbian plasticity.

### The need for competition

Theoretical analyses have demonstrated that the establishment and refinement of sensory representations during development (Wiesel and Hubel, 1963; Aitkin et al., 1970; Merzenich et al., 1975; Thompson et al., 1983; Feldman, 2009) requires strong competition between projecting fibers. An increase of the weights of synapses formed by some fibers takes place at the expense of synapses formed by other fibers, which must decrease their weights (von der Malsburg, 1973; Miller and MacKay, 1994; van Ooyen, 2001). In the mature brain, synaptic competition is instrumental, for example, in learning tasks that involve discrimination (Skorheim et al., 2014). Competition between synapses, for example for limited available resources, could also be one of the natural ways in which the magnitude of possible weight change is restricted, thus preventing excessive increases of synaptic strengths. Early theoretical studies had already suggested that competition may involve a broad range of physiologically-restricted resources, such as receptor molecules, surface area, energy resources or plasticity factors (von der Malsburg, 1973; Oja, 1982).

Homosynaptic plasticity can change synaptic weights in both directions, and thus can introduce a certain degree of competition. However, this competition is restricted to synapses which are subject to specific patterns of presynaptic activation, and is determined by presynaptic activity patterns which may be independent of each other. For example, inputs expressing high frequency activity will be potentiated, and those consistently active at low frequencies will be depressed. In the framework of STDP, synapses from presynaptic neurons that are repeatedly active shortly before the postsynaptic spikes and thus fall into potentiation window will be strengthened, while those repeatedly active shortly after postsynaptic spikes, during the depression window, will be weakened. Such scenarios, though possible in theory, impose strict requirements on the patterns of input activity and their relationship to the details of plasticity rules for potentiation and depression. Moreover, in both cases, synaptic changes are restricted to active synapses. Because bidirectional changes rely on specific patterns of presynaptic activity, this mechanism represents competition between external activation patterns, but has no relation to biologically-plausible forms of cell-intrinsic competition, such as competition between synapses of the same cell for limited resources (e.g., Frey and Morris, 1997, 1998; Elliott and Shadbolt, 2002; Fonseca et al., 2004). This latter point is important because models in which competition results from implementing an underlying physiological mechanism have stronger explanatory and predictive power than those with competition imposed as a mathematical convenience (van Ooyen, 2001; Elliott and Shadbolt, 2002). Therefore, ideally, competition should be a consequence of the learning rule and not require explicit additional rules (Gerstner and Kistler, 2002).

Normalization introduces competition that is not restricted to activated synapses, because any change of synaptic weights induced by associative plasticity at a group of active synapses is accompanied by an opposite-direction change of the weights of all other synapses. Multiplicative and subtractive normalization introduce a different degree of competition between synapses, and thus may lead to different final distributions of synaptic weights and distinct functional connectivity (Miller and MacKay, 1994; Miller, 1996). For example, in a model of development of the visual cortex, multiplicative normalization leads to the development of receptive fields with a “graded” distribution of the inputs, such that most of the inputs that expressed correlated activity during the training period were represented. In contrast, with subtractive normalization the final receptive fields were restricted to a subset of inputs which expressed the strongest correlation while other inputs to a cell, including those weakly correlated, were eliminated (Miller and MacKay, 1994).

To summarize, theoretical analysis clearly shows the necessity of mechanism(s) that (i) counteract positive feedback imposed by Hebbian-type rules on synaptic weights and neuronal activity and prevent their runaway dynamics, and (ii) introduce synaptic competition. It has also identified the following features of candidate mechanisms. First, such mechanisms should be able to robustly support the stability of network operation under a broad range of conditions, such as experimentally observed variability in the details of plasticity rules, and diverse patterns of activity. Second, it should prevent runaway dynamics of synaptic weights, but also support synaptic competition. It is not clear if both roles are related and served by a single mechanism, such as the normalization, or mediated by diverse mechanisms. Third, candidate mechanisms should include heterosynaptic plasticity, because changes of synaptic weights at non-active synapses seem to be necessary for achieving stable dynamics of model neurons and networks with plastic synapses. Finally, it should operate on a timescale comparable to the timescale of Hebbian-type synaptic plasticity. This latter requirement would be automatically fulfilled if homeostatic mechanism(s) in question and Hebbian-type plasticity share the trigger and are mediated by overlapping intracellular machinery.

## Biological candidate: homeostatic synaptic scaling

### Experimental phenomena

A form of plasticity which has received much attention as a potential mechanism of stabilization of neuronal activity is the phenomenon of homeostatic synaptic scaling (Turrigiano et al., 1998; recently reviewed in Burrone and Murthy, 2003; Turrigiano and Nelson, 2004; Rich and Wenner, 2007; Watt and Desai, 2010; Turrigiano, 2012). Homeostatic synaptic scaling is defined as compensatory up- or down- scaling of synaptic weights triggered by prolonged dramatic changes of neuronal activity, whereby synaptic weights adjust to counteract changes of activity. Synaptic weights scale up after hours and days of activity blockade by tetrodotoxin (TTX; Turrigiano et al., 1998) or hyperpolarization of neurons caused by expression of inwardly rectifying potassium channels (Burrone et al., 2002), and scale down after prolonged increases of activity by blockade of inhibition (Turrigiano et al., 1998). This scaling is multiplicative and thus maintains the relative strength of existing synaptic weights. Because homeostatic synaptic scaling is triggered by changes of neuronal firing and acts to neutralize these changes, it can be considered a mechanism of firing rate homeostasis. Originally discovered in dissociated neuron cultures, scaling has been also demonstrated in the whole brain after hours of deafferentation (Becker et al., 2013; Keck et al., 2013; Vlachos et al., 2013). Homeostatic synaptic scaling in the neocortex is developmentally regulated, such that layer 4 neurons express a transient ability to scale, and subsequently neurons from layers 2/3 demonstrate the phenomena which persist through adulthood (Turrigiano, 2012).

Although originally homeostatic scaling was described as a global cell-wide process, later studies raised the possibility that scaling could be quasi-local (at the spatial resolution of dendritic branches) which could serve for localized normalization of weights in a computationally useful way (Burrone et al., 2002; Branco et al., 2008; Rabinowitch and Segev, 2008). Realistically homeostatic scaling is not a unitary phenomenon, but involves a multitude of synaptic mechanisms such as scaling of synaptic weight at the postsynaptic site (Turrigiano et al., 1998) and changes of release probability (Bacci et al., 2001; Murthy et al., 2001; Thiagarajan et al., 2005). Homeostatic regulation of synaptic drive may be also achieved with nonsynaptic mechanisms such as changes of the intrinsic excitability of neurons (Zhang and Linden, 2003; Karmarkar and Buonomano, 2006). Possible triggers, in addition to originally suggested changes of the postsynaptic firing rate, include changes in transmitter release or activation of postsynaptic receptors, and changes of calcium influx (Burrone and Murthy, 2003; Hou et al., 2008; Fong et al., 2015). A common feature of the events which activate homeostatic plasticity is their long duration, hours and days, and extreme magnitude, such as complete blockade of activity or elimination of sensory input after peripheral lesions, or substantial increase of activity induced by a blockade of inhibition. The activated mechanisms are aimed to counteract the effects of these changes on neuronal activity and push the firing rate back to a set point.

Homeostatic synaptic scaling, operating alongside other plasticity mechanisms, has an established role in the compensatory plastic changes observed in the visual cortex in response to dramatic distortions of normal development, such as ocular dominance plasticity in experimental paradigms of monocular deprivation (Desai et al., 2002; Watt and Desai, 2010; Vitureira et al., 2012; Keck et al., 2013; Lambo and Turrigiano, 2013). It may also be involved in maintaining activity level during normal development, especially in periods of synaptogenesis and pruning (Desai et al., 2002; Turrigiano, 2012). It has been proposed to contribute to the maintainance of normal patterns of sleep oscillations after thalamic lesions (Lemieux et al., 2014). However, while synaptic scaling may be suited to adjust long term and drastic alterations of activity, and supplement other homeostatic mechanisms operating during development, two features make it a poor candidate for serving the acute constraining role necessary to combat runaway dynamics imposed by Hebbian-type plasticity rules: the time scale and the trigger for induction.

### Timescale of homeostatic synaptic scaling and runaway dynamics

Homeostatic synaptic scaling operates on the timescale of hours and days. The “rapid” scaling takes place after 4 h of complete silencing of cultured neurons with TTX (Ibata et al., 2008). This timescale is compatible with that of developmental processes, such as the formation of sensory representations in norm and pathology (Wiesel and Hubel, 1963; Aitkin et al., 1970; Merzenich et al., 1975; Thompson et al., 1983; Feldman, 2009), but it is at least two orders of magnitude slower than the timescale of associative plasticity, which changes synaptic weights within minutes or even tens of seconds. Runaway dynamics of synaptic weights and activity can be induced by Hebbian-type learning rules within seconds or minutes (e.g., Chen et al., 2013; Zenke et al., 2013). Mechanisms of homeostatic synaptic scaling will be engaged and start affecting synaptic weights after a system has been in a runaway state for hours, which prevents its normal operation. Because of this fundamental difference of the timescales, homeostatic synaptic scaling cannot mediate synaptic competition or normalize synaptic weights during on-going associative synaptic plasticity. For the same reason, it is also not suitable for counteracting runaway dynamics induced by associative plasticity. This inconsistency between time scales of slow homeostatic scaling and fast associative learning has been pointed out by Wu and Yamaguchi (2006) who concluded that synaptic scaling does not seem to work for fast learning. A recent theoretical study confirmed this conclusion, demonstrating that for achieving robust stability of a system with Hebbian-type plastic synapses, the mechanism that maintains homeostasis and prevents runaway dynamics must operate on a time scale comparable to the plasticity itself (Zenke et al., 2013).

### Realism of experimental paradigm: trigger for homeostatic synaptic scaling

One further concern regarding possible involvement of homeostatic synaptic scaling in balancing synaptic changes induced by Hebbian-type plasticity is the severity of changes that are required to trigger the scaling. Typically, scaling-up is induced by a complete silencing of activity for many hours by TTX application (Turrigiano et al., 1998; Turrigiano, 2012). Such dramatic and global changes of activity are neither likely, nor compatible with normal operation of the brain. A recent study demonstrated that 6 h after complete bilateral retinal lesions, activity in the visual cortex is reduced to ~60% of pre-lesion level (Keck et al., 2013). The authors did see evidence for homeostatic synaptic scaling after the lesions, but noted that homeostatic scaling alone could not explain the observed recovery of activity in the deprived cortex (Keck et al., 2013). Note that even this more “modest” intervention represents extreme pathology. In contrast, Hebbian-type synaptic plasticity can be induced by far more subtle events (e.g., see Chistiakova and Volgushev, 2009 for number of spikes in typical plasticity-induction protocols), and activity changes that may result from associative synaptic plasticity might be also far less dramatic.

In theory, the requirement for a dramatic and prolonged change of activity for triggering the homeostatic synaptic scaling and its very long time scale could be related issues: if the rate of change is very low, it will take a long time until a change becomes detectable. Indeed, in experiments on cultured neurons, the amplitude of miniature EPSCs increased progressively during TTX application (e.g., Turrigiano et al., 1998; Ibata et al., 2008). Averaged rates of change in the amplitudes of miniature EPSCs calculated from these experiments were at ~2% increase per hour during complete blockade of spiking with TTX, and ~0.6% decrease per hour during bicuculline-induced increase of activity. Note that thousands to tens of thousands of “extra” spikes were generated during hours of elevated activity, or were missing during hours of reduced activity. Existing experimental evidence neither provides proof, nor allows us to exclude the possibility that homeostatic synaptic scaling can indeed be triggered by physiological-range changes of activity. It is clear, however, that even if it is induced by physiologiocal changes of activity, these changes are both too small and too slow to be able to counteract tendency for runaway dynamics induced by Hebbian-type learning.

### Computational properties of homeostatic synaptic scaling

Depending on the pattern of changes of the input, homeostatic synaptic scaling may have diverse effects on the operation of neuronal networks: the per design homeostatic effect on activity, a normalizing effect on synaptic weights and normalization-related competition, but also a destabilizing effect on synaptic weights and neuronal activity.

Normalization of synaptic weights by the mechanism of homeostatic synaptic scaling can be understood as following. Consider a simplistic situation in which postsynaptic firing of a neuron is proportional to its total synaptic drive. Potentiation of a portion of synapses would lead to a firing rate increase. To counteract this increase, a mechanism of firing rate homeostasis would scale down all synapses to restore the target firing rate, and therefore also the total synaptic drive, thus performing multiplicative normalization of synaptic weights. This effect might be considered a “delayed normalization,” as the feedback from a change in activity to synaptic scaling operates via the slow loop of firing rate homeostasis. The time scale of this delayed normalization is determined by the time scale of homeostatic scaling. To attain in computer models normalization-derived properties such as synaptic competition or prevention of runaway dynamics of activity, the homeostatic scaling is often implemented on a relatively short time scale of seconds or minutes. However, these models might not reflect computational properties of experimentally observed homeostatic synaptic scaling, because reported timescale is at least two orders of magnitude longer (hours and days). In fact, we are not aware of a computational study in which homeostatic synaptic scaling with experimentally-observed features, specifically the requirement of at least 4 h of altered activity level to produce observable synaptic changes, was shown to be effective in preventing runaway dynamics or supporting synaptic competition during on-going learning.

One further factor that limits the relevance of homeostatic synaptic scaling for maintaining the normal operation of neuronal networks is that this kind of plasticity is triggered by lasting and drastic changes of overall activity, such as a persistent increase or decrease of the firing rate. In biological neuronal networks, the level of activity is subject to energy constraints and is tightly controlled by diverse fast-scale mechanisms operating at both the network and at the cellular levels. At the network level, activity is controlled by inhibition, including recurrent inhibition, which by design limits both the magnitude and the duration of episodes of elevated activity. Strong recurrent inhibition mitigates changes of activity level even when external input changes dramatically, allowing neuronal networks operate in a regime of dynamically balanced excitation and inhibition (Wehr and Zador, 2003; Okun and Lampl, 2008; Ozeki et al., 2009; Dorn et al., 2010; Sun et al., 2010). For example, complete binocular retinal lesions which resulted in an extreme change of the afferent input to the visual cortex, led to only a ~40% reduction of activity in visual cortex of the mouse (Keck et al., 2013). Moreover, inhibitory plasticity can adjust the strength of inhibition and maintain the excitatory/inhibitory balance in neurons and neuronal networks (Vogels et al., 2011; Luz and Shamir, 2012).

At the level of synapses, mechanisms regulating the input strength on a fast time scale include short-term plasticity, vesicle recycling and fast retrograde signaling. Episodes of strong presynaptic activity lead to depletion of the ready-to-release pool of vesicles, thus limiting release during the following seconds and minutes, or setting a new, lower, steady state of release (Abbott et al., 1997; Tsodyks and Markram, 1997; Varela et al., 1997, 1999; Markram et al., 1998; Sussillo et al., 2007; Costa et al., 2013). Episodes of strong postsynaptic firing and depolarization lead to activation of retrograde signaling that reduces transmitter release (Pitler and Alger, 1992; Wilson and Nicoll, 2001; Freund et al., 2003; Hashimotodani et al., 2007). Strong pre and postsynaptic activity is associated with the release of adenosine and cyclic adenosine-phosphates from neurons and glial cells and thus the elevation of extracellular adenosine levels in a local area where active synapses and neurons are located (Pascual et al., 2005; Wall and Dale, 2008; Halassa et al., 2009; Lovatt et al., 2012). Because adenosine has a suppressive effect on synaptic transmission in the neocortex and hippocampus (e.g., Dunwiddie and Haas, 1985; Scanziani et al., 1992; Thompson et al., 1992; Kerr et al., 2013; Bannon et al., 2014; Zhang et al., 2015), this would hinder further buildup of activity at this location. These are just few examples of mechanisms which operate on a substantially faster scale than homeostatic synaptic scaling and which might effectively combat excessive lasting changes of activity and restore activity level long before homeostatic synaptic scaling is activated.

The long time scale of homeostatic synaptic scaling is compatible with the time scale of developmental processes, or compensatory processes during recovery from injury. Homeostatic synaptic scaling may play a role in maintenance of overall activity level during normal development, especially in periods of synaptogenesis and pruning (Desai et al., 2002; Turrigiano, 2012). It may also play a role in pathological conditions, when the mechanisms which maintain the activity level during normal operation, are impaired or overloaded and cannot cope with drastic changes of activity caused by pathology. Indeed, evidence for homeostatic synaptic scaling has been reported in the visual cortex after binocular retinal lesions (Keck et al., 2013) and in the dentate gyrus after denervation (Becker et al., 2013; Vlachos et al., 2013). However, in pathological conditions the “homeostatic” synaptic scaling may also have a destabilizing effect on synaptic weights and neuronal activity. For example, if activity is reduced temporarily (e.g., because of a reversible injury to a peripheral sensory apparatus), the up-scaling of synaptic weights could lead to over-excitability of neurons when input firing recovers. Thus, because synaptic up-scaling follows activity changes with a delay of several hours, it may lead to an over-shoot of activity when the input is recovering. Indeed, network models of pathological conditions, lesions or deafferentation, show that homeostatic synaptic scaling helps to recover normal activity patterns after small and moderate deafferentation, but leads to post-traumatic seizures if the degree of deafferentation is above a certain threshold (about 80%; Houweling et al., 2005; Fröhlich et al., 2008; Volman et al., 2011a,b). One of the contributing mechanisms here may be the formation of new silent synapses during prolonged silencing of activity, which leads to enhancement of LTP induction (Arendt et al., 2013). If a similar process takes place after cortical damage, potentiation of these new synapses after partial recovery of the activity will further amplify the increase of the overall synaptic drive, which might facilitate the development of seizures or other pathological activity patterns.

To summarize, homeostatic synaptic scaling represents a set of mechanisms which are triggered by extreme and long lasting (hours and days) changes of neuronal activity, and serve to counteract firing rate changes by up- or down-scaling synaptic weights. These mechanisms operate on time scales which are orders of magnitude longer than the time scale at which associative plasticity is induced. Therefore, they would not be engaged or expressed until runaway dynamics had created an unstable and saturated network, which generates dramatically altered activity for hours. Thus, experimentally observed properties of homeostatic synaptic scaling do not fit two crucial requirements for a hypothetical mechanism which maintains stability of operation and provides synaptic competition in systems with Hebbian-type learning rules. Both the trigger and the time scale of synaptic scaling are fundamentally different from those of the Hebbian-type plasticity.

## Biological candidate: heterosynaptic plasticity

### Experimental phenomena

Heterosynaptic plasticity refers to changes at synapses which were not active during the induction of plasticity (Figure 1). Heterosynaptic LTD accompanying the induction of LTP was first described in the hippocampus shortly after the phenomenon of LTP was discovered (Lynch et al., 1977). In CA1 pyramidal neurons, induction of LTP of Schaffer collateral-commissural synapses at apical dendrites was accompanied by LTD at inputs to basal dendrites made by commissural fibers that were not stimulated during the induction (Figure 1A, left). Vice versa, induction of LTP at the basal dendrites was accompanied by LTD at the apical dendrites. Heterosynaptic LTD accompanying the induction of homosynaptic LTP clearly has potential for both balancing plastic changes and supporting synaptic competition.

Spatial distribution of LTP and LTD studied in structures with a regular organization of their inputs, such as the hippocampus or amygdala, revealed a bi-phasic Mexican-hat type profile (White et al., 1990; Royer and Paré, 2003). Induction of LTP at a set of synapses was accompanied by a weaker heterosynaptic LTP at nearby inputs, and heterosynaptic LTD at more distant inputs (Figure 1A, right). A symmetrical profile of heterosynaptic changes was observed around the site of LTD induction: weaker LTD at close distances and LTP at more distant inputs (Royer and Paré, 2003). Because the amount of potentiation and depression in these profiles was balanced, this type of heterosynaptic plasticity can provide a powerful local mechanism of both normalization of synaptic weights and synaptic competition.

In the CA1 region of the hippocampus, pairing of one input to a pyramidal neuron led to potentiation not only of that stimulated synapse, but also of synapses formed by nearby fibers on that neuron, and even on nearby neurons (Bonhoeffer et al., 1989; Kossel et al., 1990; Schuman and Madison, 1994; Engert and Bonhoeffer, 1997).

This evidence for heterosynaptic plasticity indicates that presynaptic activation of the synapse is not a strict requirement for plasticity induction. Indeed, long term plasticity can be induced by purely postsynaptic protocols. In the hippocampus and neocortex, photolysis of caged Ca^2+^ (Neveu and Zucker, 1996a,b; Yang et al., 1999) or postsynaptic spiking (Kuhnt et al., 1994; Volgushev et al., 1994, 2000; Cummings et al., 1996; Chistiakova and Volgushev, 2009; Lee et al., 2012) is sufficient to induce plasticity.

### Trigger for heterosynaptic plasticity

Heterosynaptic changes are triggered by acute rises of intracellular Ca^2+^ concentration (Yang et al., 1999; Balaban et al., 2004; Lee et al., 2012), thus sharing the trigger with Hebbian-type plasticity (Lisman, 1989; Artola and Singer, 1993; Cummings et al., 1996). The required rises of [Ca^2+^] can be produced by bursts of action potentials backpropagating throughout the dendritic tree (Spruston et al., 1995; Staubli and Ji, 1996; Larkum et al., 1999; Golding et al., 2002; Waters et al., 2003; Lisman and Spruston, 2005; Sjöström and Häusser, 2006; Remy and Spruston, 2007). Chelation of intracellular calcium impairs induction of heterosynaptic plasticity (Lee et al., 2012). In addition to the shared calcium dependence, intracellular mechanisms of of homosynaptic and heterosynaptic plasticity overlap, as indicated by at least partial occlusion between homo- and hetero-synaptic plastic changes (Kuhnt et al., 1994; Cummings et al., 1996; Neveu and Zucker, 1996a,b; Volgushev et al., 1999; Yang et al., 1999). Thus, heterosynaptic plasticity is induced by the same protocols, occurs at the same timescale, and shares mechanisms with Hebbian-type plasticity (see below, **Figure 5** and related text for further discussion).

### Properties of heterosynaptic plasticity

**Figure 4:**
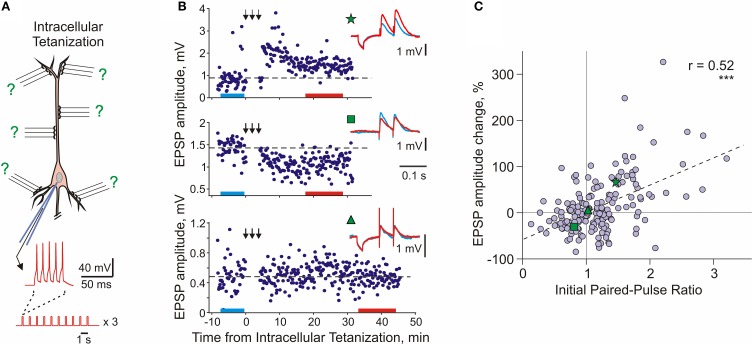
**Long-term synaptic plasticity induced by intracellular tetanization. (A)** A scheme of an intracellular tetanization experiment. Bursts of short depolarizing pulses (5 pulses at 100 Hz; 10 bursts at 1 Hz, 3 trains of 10 bursts) were applied through the recording electrode without presynaptic stimulation to induce bursts of action potentials. Synaptic responses were recorded before and after the intracellular tetanization. Because no inputs were stimulated during the induction, plasticity at all synapses can be considered heterosynaptic. **(B)** Examples of inputs that underwent potentiation (top), depression (middle), or did not change (bottom) after intracellular tetanization in pyramidal neurons from slices of rat visual cortex. Time courses of amplitudes of EPSPs evoked by the first pulse in a paired-pulse paradigm. The timing of intracellular tetanization is indicated by the arrows above each plot. Insets show averaged responses to paired pulse stimuli before and after intracellular tetanization, from color-coded time intervals. In this example, LTP and LTD were induced simultaneously at two inputs to the same neuron (top and middle). Note that input resistance of neurons measured by responses to small hyperpolarizing pulses applied before synaptic stimuli remained unchanged. **(C)** Correlation between changes of EPSP amplitude after intracellular tetanization and initial paired-pulse ratio. Data for *N* = 136 inputs to pyramidal neurons in slices of visual cortex (*N* = 60 inputs) and auditory cortex (*N* = 76 inputs). Green symbols (star, square, and triangle) refer to the example inputs from **(B)**. (Modified, with permission, from Chen et al., 2013).

Heterosynaptic, long-term plastic changes can be induced in hippocampal and neocortical neurons by intracellular tetanization—bursts of spikes evoked by short depolarizing pulses applied through the recording electrode (Figure 4A; Kuhnt et al., 1994; Volgushev et al., 1994, 1997, 1999, 2000; Chistiakova and Volgushev, 2009; Lee et al., 2012). The rationale behind the intracellular tetanization protocol as a tool to study heterosynaptic plasticity is the following. Each neuron in the neocortex receives thousands of synaptic inputs, but activation of only a fraction of these inputs, few dozens to hundreds, is necessary to evoke spikes. Repetitive activation of a fraction of inputs and repetitive firing of the postsynaptic cell can, under certain conditions, induce synaptic plasticity. During the induction, all synapses but for those of the activated fraction will experience postsynaptic activity without activation of their presynaptic fibers. This situation, postsynaptic activity without presynaptic activation, is mimicked by the intracellular tetanization (Figure 4A). Because none of the synaptic inputs was stimulated during the intracellular tetanization, any changes of synaptic transmission after the intracellular tetanization can be considered heterosynaptic. It is important to note that postsynaptic activity during intracellular tetanization (~150 spikes) is both compatible with activity patterns observed *in vivo*, for example during visual stimulation (e.g., Volgushev et al., 2003), and comparable to postsynaptic activity during typical plasticity-induction protocols (see Chistiakova and Volgushev, 2009 for comparison of number of spikes in plasticity-induction protocols).

Following intracellular tetanization, amplitudes of synaptic responses could increase, decrease or not change (Figure 4B). The amplitude changes occurred fast, on the same time scale as homosynaptic changes. Moreover, intracellular tetanization could simultaneously induce LTP and LTD in two independent inputs onto one cell (Figure 4B top and middle). The direction of plastic change of a synaptic input was correlated with the initial paired-pulse ratio, a measure which is inversely related to release probability (Figure 4C, Volgushev et al., 1997, 2000; Lee et al., 2012; Chen et al., 2013). Inputs which initially had a low release probability (high initial paired-pulse ratio) were typically potentiated. Inputs that had a high release probability (low initial paired-pulse ratio) were typically depressed or did not change. Thus, the direction of heterosynaptic changes depends on initial properties of a synapse, and is determined at each synapse individually. Weight-dependence is one further similar feature of heterosynaptic and homosynaptic plasticity: it has been also reported for LTP and LTD induced by afferent tetanization or by a pairing procedure in the hippocampus and neocortex (van Rossum et al., 2000; Sjöström et al., 2001; Hardingham et al., 2007).

The weight-dependence of heterosynaptic plasticity might reflect history-dependent predispositions of synaptic inputs to undergo potentiation or depression (Volgushev et al., 1997, 2000; Chistiakova and Volgushev, 2009). Weak synaptic inputs with low release probability, such as those which underwent depression in the past, are less susceptible to further depression yet have a stronger predisposition for potentiation. Strong synapses with a high release probability, such as those recently potentiated, have a higher predisposition for depression. The notion of the predisposition of synapses for plastic changes is closely related to the ideas of a sliding threshold between depression and potentiation in the BCM rule (Bienenstock et al., 1982; Yeung et al., 2004) and metaplasticity – history-dependent changes of the ability of synapses to undergo potentiation or depression (Abraham and Bear, 1996; Clem et al., 2008).

Thus, heterosynaptic plasticity induced by strong postsynaptic activity has properties which make it an ideal candidate for counteracting runaway dynamics of synaptic weights and mediating synaptic competition. Heterosynaptic plasticity, while not requiring presynaptic activity at the synapse for the induction, has the same trigger (rise of intracellular calcium), partially overlapping mechanisms of expression, and operates on the same time scale as homosynaptic plasticity. Moreover, heterosynaptic changes can be induced by the same protocols which are typically used to induce homosynaptic plasticity.

### Heterosynaptic plasticity in published studies: meta-analysis

**Figure 5:**
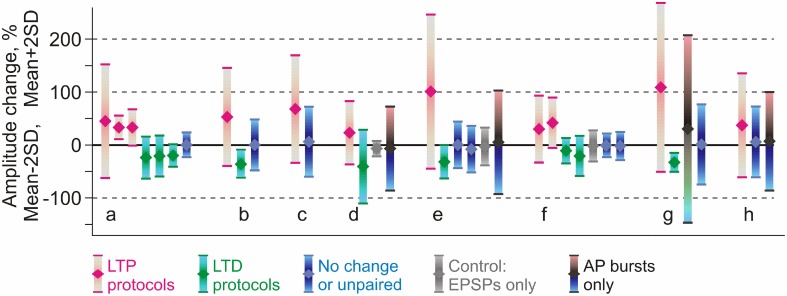
**Comparison of reported changes of response amplitude at inputs that were active during the induction (homosynaptic, input-specific) and those not active during the induction (heterosynaptic)**. The plot shows results of 36 experimental series (bars) from eight papers (groups of bars) on pairing-induced long-term plasticity (STDP), in which the mean amplitude changes were reported together with the SD (or SEM) and number of observations. Each bar shows an average (diamond symbol) change of EPSP amplitude after pairing procedure ±2 SD. This range includes 95% of normally distributed values. Magenta: changes after LTP protocols (post after pre). Green: changes after LTD protocols (pre after post). Blue: range of EPSP amplitudes after protocols that did not lead to significant changes of the averaged response (such as interval between pre and post spikes outside plasticity windows). Gray: range of EPSP amplitudes after only presynaptic stimulation without postsynaptic spikes. Black, bars from cyan to pink (in d,e,h): range of EPSP amplitudes after bursts of postsynaptic spikes only, without presynaptic stimulation. Data for excitatory inputs to L2/3 or L5 pyramidal neurons from somatosensory, visual or auditory cortex, from the following papers: Feldman (2000) (a); Sjöström et al. (2001) (b); Watt et al. (2004) (c); Birtoli and Ulrich (2004) (d); Nevian and Sakmann (2006) (e); Letzkus et al. (2006) (f); Hardingham et al. (2007) (g); Chistiakova et al. (2014) (h). Results from Hardingham et al. (2007) (g) present the LTP and LTD data selected by the direction of the change. The third bar in this group shows LTP and LTD data pooled together. Details of experimental protocols can be found in original papers. (Modified, with permission, from Chistiakova et al., 2014).

This latter conclusion stays in apparent contradiction to the wealth of publications reporting that amplitude of responses in non-activated or control inputs did not change, and, more generally, to the notion of input specificity of homosynaptic plasticity. We suggest that this contradiction could be due to the fact that heterosynaptic changes are bidirectional but balanced. To test this conjecture, we re-analyzed results from eight papers on STDP of excitatory inputs to layer 2/3 or layer 5 pyramidal neurons in slices from somatosensory, visual or auditory areas of rat neocortex (Feldman, 2000; Sjöström et al., 2001; Birtoli and Ulrich, 2004; Watt et al., 2004; Letzkus et al., 2006; Nevian and Sakmann, 2006; Hardingham et al., 2007; our data from Chistiakova et al., 2014). In all of these papers clear cases of homosynaptic LTP or LTD are presented. Figure 5 illustrates the results of 36 experimental series from these papers, as the averaged change of response amplitude (diamond symbol) after the pairing or control procedure and the range covered by ±2 SD. This range includes 95% of normally distributed values, however because number of measurements contributing to each experimental series was not high, typically between *N* = 4 and *N* = 20, the actual measured values did not necessary covered the whole ±2 SD range. Figure 5 illustrates several important points. First, most “No change or unpaired” groups (Figure 5, blue) and especially “AP bursts only” groups (Figure 5, blue-gray-pink bars) have high variance, with the ranges of response amplitude changes overlapping substantially with the ranges of homosynaptic changes after LTP and LTD protocols. This implies that “No change” and “AP bursts only” groups must have contained individual cases of potentiation and depression, which were heterosynaptic LTP and LTD in experiments in which no presynaptic stimulation was applied. Moreover, because the averages were not significantly different from zero potentiation and depression were balanced. Second, although on average LTP protocols increased and LTD protocols decreased response amplitude (Figure 5, magenta and green), the effects were highly variable, often including changes in the opposite directions. Assuming continuous distributions of EPSP amplitude changes over the mean ± 2 SD range, the LTP and LTD protocols might have induced plastic changes of both signs. In 8 out of 11 LTP groups the value of mean −2 SD is well below zero, suggesting that some of the inputs were depressed. In 5 out of 9 LTD groups mean +2 SD reaches well above zero, suggesting that some inputs were potentiated. In most LTP and LTD groups (17 out of 20) there should have been inputs which did not change (Figure 5). This suggests that factors other than timing, such as synaptic predispositions for plasticity, might have contributed to the final effect of the plasticity-induction protocol on response amplitude. This conjecture is supported by the results of Hardingham et al. (2007), who found that the same protocol could induce either potentiation or depression. The direction of the EPSP amplitude change was correlated with the release probability of the synapse before the plasticity induction. This finding is corroborated by our results (Figure 4C, modified from Chen et al., 2013). Finally, the range of EPSP amplitude changes in unpaired inputs was typically smaller than the range of amplitude changes induced by spike burst-only protocols (Figure 5, d,e,h). This may reflect competition of plastic synapses for limited resources. In this scenario, pairing may facilitate access to resources for homosynaptic plasticity at paired inputs via a mechanism of synaptic tagging (Frey and Morris, 1997, 1998; Fonseca et al., 2004) or a similar process, thus leaving fewer resources available for heterosynaptic changes at unpaired inputs. Spikes-only protocols leave more resources available for heterosynaptic changes, and thus induce heterosynaptic plasticity of a larger amplitude. Note that this latter point (larger variance after spike-burst only protocols as compared to changes in un-paired inputs) is suggested by our meta-analysis (Chistiakova et al., 2014), though limited number of studies precludes statistical analysis. This question needs to be tested in future work.

The above analysis demonstrates that both potentiation and depression might have been induced in individual unpaired inputs and also by spike bursts-only protocols (Figure 5). However, when averaged across the inputs, EPSP amplitude changes were not significant because of the balanced nature of heterosynaptic plasticity. It is also important to note that in papers specifically aimed at investigating heterosynaptic plasticity, it was readily induced by regular pairing (Nishiyama et al., 2000; Huang et al., 2008; Arami et al., 2013), afferent tetanization (Cummings et al., 1996; Staubli and Ji, 1996; Chevaleyre and Castillo, 2003; Royer and Paré, 2003; Bauer and LeDoux, 2004; Pascual et al., 2005; Nugent et al., 2007; Wöhrl et al., 2007), or purely postsynaptic protocols (e.g., Pockett et al., 1990; Christofi et al., 1993; Volgushev et al., 1994, 1997, 1999, 2000; Cummings et al., 1996; Lee et al., 2012). This analysis substantiates our conclusion that induction of homosynaptic plasticity by a typical pairing procedure used in STDP studies is accompanied by induction of heterosynaptic plasticity in unpaired inputs.

To summarize, heterosynaptic plasticity induced by intracellular tetanization expresses properties that are well suited for serving as a robust mechanism of normalization of synaptic weights: (i) it depresses strong and potentiates weak synapses thus preventing runaway dynamics of synaptic weights, (ii) it is induced at non-active synapses by the same protocols which induce homosynaptic plasticity, providing for explicit competition and (iii) it operates on the same time scale as homosynaptic plasticity.

## Modeling heterosynaptic plasticity

### Heterosynaptic plasticity robustly prevents runaway dynamics

To test the hypothesis that heterosynaptic plasticity can prevent runaway dynamics of synaptic weights and activity (Volgushev et al., 2000; Chistiakova and Volgushev, 2009) we used a neuron model with synaptic weight changes governed by STDP rules and heterosynaptic plasticity with experimentally observed properties (Chen et al., 2013). The model neuron received inputs from 100 simulated presynaptic neurons, firing action potentials with Poisson distributed interspike intervals. Activity of presynaptic neurons was mildly correlated, with an averaged cross-correlation between pairs of spike trains of 0.35 ± 0.05. Averaged presynaptic firing at 1 Hz led to the firing of the postsynaptic model neuron at ~1.8 Hz. Synaptic weight changes were governed either by STDP rules (STDP-only models) or by STDP rules complemented with heterosynaptic plasticity (STDP + heterosynaptic plasticity models). Heterosynaptic plasticity was implemented according to experimental data (Volgushev et al., 2000; Chistiakova and Volgushev, 2009; Lee et al., 2012; Chen et al., 2013). It was triggered by increases of intracellular calcium concentration above a threshold level, and affected all synapses in a weight-dependent manner: the probability of synaptic change, its direction, and its magnitude depended on the initial weight. These dependences were implemented using the Equations (1) and (2).

(1) $$\text{P}=3000\times {\left({\text{W}}_{\text{syn}}-{\text{W}}_{\text{max}}/2\right)}^{2}+0.1$$

where P is the probability of the synaptic change, W_syn_ is the current synaptic strength and W_max_ = 0.03 mS/cm^2^ is the maximal synaptic strength. According to Equation (1), P is equal to 0.1 for synapses with intermediate strength, and P equals to ~0.775 for synapses with maximal or minimal strength. The change of synaptic weight dW_syn_ was calculated according to following equation:

(2) $$\begin{array}{l} {\text{dW}}_{\text{syn}}=(([1/(1+\text{exp}(({\text{W}}_{\text{syn}}-(0.5\times {\text{W}}_{\text{max}}))\times 100))]-0.5)\\ \;+\sigma \times 0.02)\times 0.0001 \end{array}$$

In this equation, dW_syn_ indicates the change of synaptic strength and σ is a random variable drawn from Gaussian distribution with zero mean and standard deviation of 3. A detailed description of the model, implementation of plasticity rules and discussion of parameters can be found in the original publication (Chen et al., 2013).

**Figure 6:**
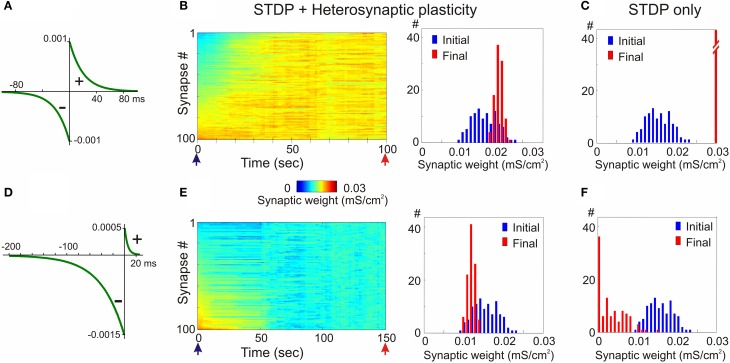
**Heterosynaptic plasticity prevents runaway dynamics produced by positively and negatively biased STDP. (A,D)** STDP rules. STDP learning rule with symmetrical potentiation and depression windows (**A**, τ^+^ = τ^−^ = 20 ms; a^+^ = a^−^ = 10^−3^ mS/cm^2^), and with negative bias (**D**, τ^+^ = 5 ms, a^+^ = 0.5 × 10^−3^ mS/cm^2^, τ^−^ = 40 ms, a^−^ = 1.5 × 10^−3^ mS/cm^2^). **(B,E)** Heterosynaptic plasticity prevents runaway dynamics of synaptic weights. Same models as in Figures 2, 3 but with the mechanism for heterosynaptic plasticity as described in Chen et al. (2013). Note that in both models, with symmetrical **(B)** and negatively-biased **(E)** STDP rules, synaptic weights are not saturated, but remain normally distributed within the operation rage. **(C,F)** For comparison, distributions of synaptic weights in STDP only models from Figures 2, 3, expressing runaway dynamics are shown. (Modified, with permission, from Chen et al., 2013).

In the first example, STDP with symmetrical windows for potentiation and depression was used (Figures 6A–C; same STDP rules as in Figure 2). In the model with the STDP-only learning rule, synaptic weights expressed clear runaway dynamics and were saturated at the maximal value by the end of a 100s simulation (Figures 2A,C, 6C). This led to a profound increase of the postsynaptic firing rate despite unchanged presynaptic firing (1 Hz throughout the simulation). The postsynaptic firing rate increased from ~1.8 Hz during the first 10 s of simulation, to ~6.3 Hz during the last 10 s of simulation. Implementing in the model heterosynaptic plasticity with experimentally observed properties in addition to STDP-rules effectively prevented runaway dynamics of synaptic weights and activity. Synaptic weights in this model increased slightly, but did not saturate. The new stable distribution of synaptic weights around the new mean value was completely located within the operational range of synapses (Figure 6B). Firing rate of the postsynaptic neuron slightly increased from ~1.8 to ~2.6 Hz.

In the second example model, the STDP-rule was strongly biased toward depression (Figure 6D, same STDP rule as in Figure 3). In the STDP-only model, synaptic weights expressed clear runaway dynamics toward the minimum value (Figures 3A,C, 6F). The decreased synaptic weights were not producing sufficient depolarization to maintain spiking of the postsynaptic neuron, therefore averaged firing rate of presynaptic neurons was increased to 2 Hz and then to 3 Hz (Figure 3A). Even with the three-fold increase of presynaptic firing rate, the postsynaptic neuron became silent. A portion of synaptic weights was saturated at the minimum value (Figures 3C, 6F). Heterosynaptic plasticity effectively prevented the runaway dynamics of synaptic weights toward zero and silencing of the cell (Figure 6E). These results demonstrate that heterosynaptic plasticity can prevent runaway of synaptic weights to either extreme.

**Figure 7:**
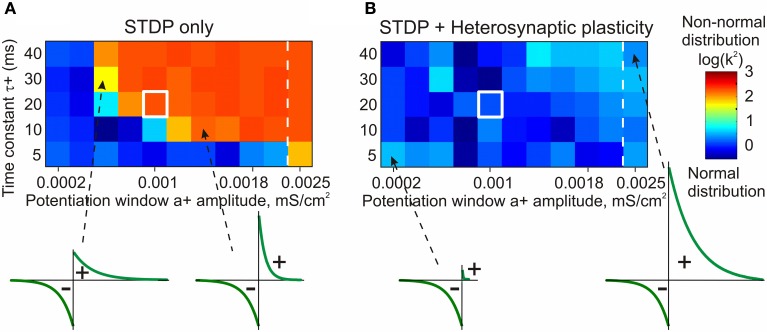
**STDP only model fails to prevent runaway dynamics of synaptic weights in broad range of STDP rules (A)**. Heterosynaptic plasticity makes a broad range of STDP parameters compatible with stable operation of neurons (**B**). Each box in the grids shows the D'Agostino-Pearson's *K*^2^-test for normality of synaptic weight distribution after 100 s of simulations with different STDP potentiation windows, with a^+^ and τ^+^ as indicated on the X and Y axes. White square indicates symmetrical STDP learning rule. Synaptic weight distributions with high *K*^2^-test values (>50) indicating deviation from normality, typically contain most of the weights saturated at maximal or minimal values. Note that in simulations with the STDP only model **(A)**, only few STDP rules, with strong bias toward depression, did not lead to runaway dynamics. Most STDP rules, including examples shown in the bottom, led to runaway dynamics of synaptic weights. In contrast, the model with STDP and heterosynaptic plasticity **(B)** did not express runaway dynamics over the whole range of tested STDP rules, including those extremely unbalanced (insets, bottom). (Modified, with permission, from Chen et al., 2013).

**Figure 8:**
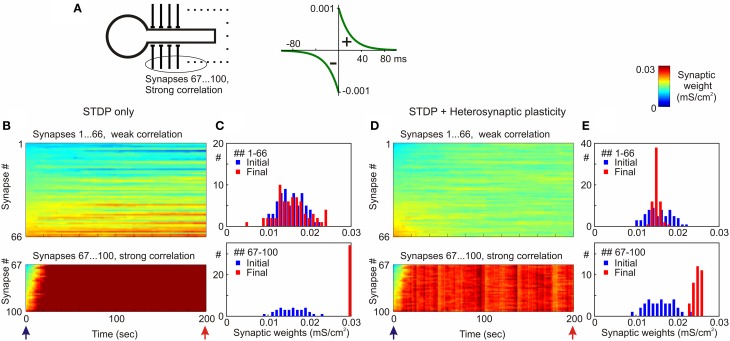
**Segregation of synaptic weights of strongly vs. weakly correlated inputs in STDP models with and without heterosynaptic plasticity. (A)** A model neuron received input from *N* = 100 presynaptic neurons firing at average frequency of 1 Hz. Spike trains of 66 presynaptic neurons (inputs # 1–66) were weakly correlated (averaged cross-correlation 0.34 + 0.02), spike trains of 34 presynaptic neurons (inputs # 67–100) were strongly correlated (averaged cross-correlation 0.61 + 0.05). Symmetrical STDP rule was used in the simulations, with τ^+^ = τ^−^ = 20 ms; a^+^ = a^−^ = 10^−3^ mS/cm^2^. **(B,D)** Dynamics of synaptic weights of weakly correlated inputs (synapses # 1…66) and strongly correlated inputs (synapses # 67…100) in the model with STDP only **(B)** and the model with STDP and heterosynaptic plasticity **(D)**. **(C,E)** Distributions of synaptic weights at the beginning (blue bars) and at the end (red) of simulations from **(B**,**D)**, respectively. Note runaway dynamics of synaptic weights and their saturation at the highest value (0.03 mS/cm^2^) for the group of strongly correlated inputs in STDP-only simulation. (Modified, with permission, from Chen et al., 2013).

The stabilizing effect of heterosynaptic plasticity on synaptic weights and activity is long-lasting and robust. Heterosynaptic plasticity was able to keep synaptic weights and activity levels within an operational range for models with different calcium thresholds for plasticity induction, models subject to different patterns of presynaptic activity, and over a broad range of parameters of STDP learning rules. This latter point is illustrated in Figure 7. To explore how changing the parameters of STDP rules affects the stability of operation of the model neuron, we systematically varied the amplitude and the time constant of the potentiation window of STDP, while keeping the depression window constant. The set of tested STDP rules covered a range from those strongly dominated by depression, to those with balanced potentiation and depression windows, as well as those dominated by potentiation (see insets in Figure 7). As an indicator of runaway dynamics we used the deviation from normality (D'Agostino-Pearson's *K*^2^-test) of synaptic weight distribution after 100 s of simulation. Note that the shape of the final distribution of synaptic weights is determined by a multitude of factors operating at each synapse. These factors include, but are not restricted to, initial synaptic weight, the set of plasticity mechanisms operating at a synapse and the specifics of these mechanisms, and the pattern of presynaptic activity experienced by a synapse (see below, Figure 8). Experimentally measured distributions of synaptic weights, usually asymmetrical and close to log-normal (e.g., Song et al., 2005), might reflect the broad variety of unique combinations of these factors at individual synapses. Because in each simulation presented here these factors were similar for all synapses, synaptic weights converged to the same value. Convergence of synaptic weights to a value within the operation range resulted in a normal distribution, while convergence to one of the extremes resulted in a distribution which deviated from normality. Therefore, we used a test of normality and deviation from normality of the final distribution of synaptic weights as an indicator of runaway dynamics.

The STDP-only model expressed non-saturating behavior only with a limited sub-set of tested STDP rules, in which the window for potentiation was smaller than the depression window (Figure 7A, blue area of the matrix). As soon as the potentiation window of the STDP rule was ~75% of the depression window or stronger, synaptic weights and postsynaptic firing invariably expressed runaway dynamics (Figure 7A, orange/red area of the matrix). In fact, the range of STDP windows compatible with stable dynamics of synaptic weights was somewhat overestimated in these experiments. In cases with a strongly dominating window for depression, the synaptic drive was reduced below the level necessary to evoke postsynaptic spiking before synaptic weights were saturated. After the postsynaptic spiking had ceased, synaptic weights did not change any more per STDP design. Addition of heterosynaptic plasticity to the model robustly prevented runaway dynamics over the whole range of tested STDP parameters, from almost exclusively depressing STDP rules, to those strongly dominated by potentiation (Figure 7B). Joint action of STDP and heterosynaptic plasticity led to the stable distribution of synaptic weights within their operational range. The new equilibrium point of the synaptic weight distribution depended on the relative strength of potentiation and depression windows: in models equipped with a stronger potentiation window of STDP the final distributions of synaptic weights were shifted toward higher values. Notably, heterosynaptic plasticity, by preventing runaway dynamics of synaptic weights, also kept averaged firing rate around the operating point (Chen et al., 2013).

Thus, heterosynaptic plasticity with experimentally observed properties provides a robust stabilizing mechanism, which makes possible the stable operation of neurons expressing a broad range of STDP parameters. This is an important feature because experimental evidence indeed shows wide variations of STDP windows for potentiation and depression and of their relative strength in neurons and synaptic connections of different types (Abbott and Nelson, 2000; Nishiyama et al., 2000; Sjöström et al., 2001; Froemke et al., 2005; Zhou et al., 2005; Haas et al., 2006; Caporale and Dan, 2008; Feldman, 2009).

### Heterosynaptic plasticity permits segregation of inputs and supports competition

Despite its strong stabilizing effect, heterosynaptic plasticity does not prevent segregation of the weights of synapses which have diverse properties or are subject to diverse input patterns, and supports synaptic competition. In the examples illustrated in Figure 8, inputs to the model neuron were segregated into two groups, one with a high and the other with a low correlation of presynaptic firing. In the STDP-only model, inputs that were strongly correlated were rapidly potentiated and saturated at the maximum value, while the weights of weakly correlated inputs changed little (Figures 8B,C). In the model with both forms of plasticity, STDP and heterosynaptic, inputs from both groups remained unsaturated. The groups of weakly and strongly correlated inputs formed two clearly separate distributions, both within the operational range of synaptic weights (Figures 8D,E; Chen et al., 2013). Similarly, segregation of synaptic weights was observed when the two groups of inputs differed by their average firing frequency rather than by their correlation. Examples from Figures 6, 8 illustrate that in the model with both forms of plasticity, STDP and heterosynaptic, the location of the steady-state distribution of a group of synaptic weights depends on the balance of several factors, such as the specifics of plasticity rules at these synapses (Figure 6), the level of correlation of presynaptic firing (Figure 8) or firing frequency.

The origin of synaptic competition arising from heterosynaptic plasticity can be understood as following. Heterosynaptic plasticity triggered by episodes of strong postsynaptic activity pushes all synapses, including those not recently activated, toward an equilibrium point. Because heterosynaptic plasticity is triggered by the same episodes of activity which induce homosynaptic changes, the induction of homosynaptic potentiation or depression does not simply push activated synapses toward the maximum or minimum, but also pushes all non-active synapses toward a separate equilibrium point. The existence of two different target weights for active vs. inactive inputs creates a contrast of forces which drive weight changes at active vs. non-active synapses. This facilitates the segregation of weights of differentially active synapses by plasticity-inducing episodes of postsynaptic activity. By driving synaptic weights toward an equilibrium point within the operating range, heterosynaptic plasticity also prevents their saturation, and supports ongoing differentiation of the weights of synapses which experience different activity. For example, if an initially large number of synapses were active in synchrony and were potentiated, but later on only a portion of them remained consistently active, the background level of competition provided by heterosynaptic plasticity would be able to suppress the remaining synapses, thus allowing for selection of only the relevant group—a process that may mediate the differentiation stage of learning. In this scenario, synapses compete for maintaining their weights at increased or decreased values set by homosynaptic plasticity, but will be driven to the heterosynaptic equilibrium point if other synapses, but not themselves, are active.

Thus, heterosynaptic plasticity facilitates segregation and competition between groups of synaptic inputs exhibiting diverse properties, such as the frequency or correlation of presynaptic firing, or details of plasticity rules. Moreover, it helps to preserve the ability of a neuron with plastic synapses for further learning: unsaturated synapses have a higher potential for further changes than those potentiated to the maximum or depressed to zero by STDP-only learning rules.

To summarize, heterosynaptic plasticity with experimentally observed properties is a strong candidate mechanism for counteracting the runaway dynamics which is imposed on synaptic weights and activity by the positive feedback of Hebbian-type learning rules. It robustly prevents runaway dynamics over a broad range of activity patterns and details of Hebbian-plasticity rules, such as the balance of STDP windows for potentiation and depression. Heterosynaptic plasticity does not prevent segregation of synaptic weights, and can support synaptic competition. Moreover, it shares the trigger, has overlapping mechanisms and operates on the same time scale as Hebbian-type plasticity. This combination of features makes heterosynaptic plasticity an ideal candidate mechanism of homeostatic control of synaptic weight changes.

## Biological candidates: other mechanisms counteracting runaway

Several further mechanisms may contribute to counteracting the tendency for runaway of synaptic weights and activity. One is saturation of plasticity: in a series of potentiation-inducing tetanizations, the magnitude of the response increase after each subsequent tetanization is diminished until the ability for further potentiation is eventually lost altogether (Colino et al., 1992; Huang et al., 1992). Another mechanism is weight-dependence of plasticity, whereby the magnitude of potentiation is smaller at strong synapses which likely were already potentiated, than at weak synapses, which did not experience prior potentiation or were previously depressed (van Rossum et al., 2000; Sjöström et al., 2001; Hardingham et al., 2007). One further mechanism is a sliding calcium threshold for potentiation and depression, whereby depending on the history of recent activity and synaptic changes, the thresholds for potentiation and depression or intracellular calcium homeostasis change (Bienenstock et al., 1982; Yeung et al., 2004). These notions and mechanisms contribute to the concept of metaplasticity—history-dependent changes of the ability of synapses to undergo further plastic changes (Abraham and Bear, 1996; Clem et al., 2008). These mechanisms are inherent to Hebbian-type plasticity rules, and thus are ideally suited to shape the ability of synapses to change. By imposing negative feedback on homosynaptic plastic changes, they clearly can limit the runaway tendency, and thus decrease the instability of a system with plastic synapses. A drawback of these mechanisms, as of any mechanisms governing homosynaptic plasticity, is that they require presynaptic activation and cannot affect inactive synapses. This requirement limits the ability of these mechanisms to serve as regulators of global, cell-wide synaptic homeostasis.

A family of non-synaptic mechanisms regulating intrinsic excitability of neurons is not restricted to activated synapses and thus does not have this limitation. These mechanisms can change the excitability of an activated dendritic branch or a whole neuron, and thus affect all respective synapses (Bliss and Lomo, 1973; Daoudal et al., 2002; Zhang and Linden, 2003; Frick et al., 2004; Karmarkar and Buonomano, 2006; Fink and O'Dell, 2009; Sehgal et al., 2013). Excitability changes may counteract synaptic changes, thus having a homeostatic effect (Zhang and Linden, 2003; Karmarkar and Buonomano, 2006), or enhance and amplify synaptic changes, thus having an anti-homeostatic effect (Frick et al., 2004; Fink and O'Dell, 2009; see Sehgal et al., 2013 for recent review).

Several mechanisms may counteract the development of runaway activity, even in cases in which runaway potentiation or depression of individual synaptic weights had not been prevented. Short-term plasticity determines transient changes in transmitter release occurring on the temporal scale from milliseconds to several seconds (Zucker and Regehr, 2002), and thus is an important factor shaping synaptic responses to sequences of presynaptic spikes (Abbott et al., 1997; Markram et al., 1998; Abbott and Regehr, 2004; Richardson et al., 2005; Sussillo et al., 2007; Costa et al., 2013). Long-term plasticity is partially expressed presynaptically and thus alters short-term plasticity (e.g., Bekkers and Stevens, 1990; Markram and Tsodyks, 1996; Schulz, 1997; Volgushev et al., 1997), affects synaptic responses to sequences of spikes, and the amplitude of steady-state responses to repetitive presynaptic spikes (Markram and Tsodyks, 1996). Because depletion of synaptic vesicles and short-term depression of transmitter release are proportional to release probability, increase of release probability in association with LTP would lead to a stronger short-term depression, while a decrease of release probability associated with LTD would lead to a weaker short-term depression. As a result, changes of steady-state synaptic responses (and thus of synaptic drive of the postsynaptic neuron) resulting from sequences of presynaptic spikes will be less pronounced than the potentiation or depression of responses to single spikes. The magnitude of this attenuating effect will strongly depend on the relative contribution of presynaptic mechanisms to LTP/LTD expression, time constants of short-term facilitation and depression in a synapse, and on presynaptic firing rate. For example, let us assume that 50% of LTP or LTD magnitude is expressed presynaptically as an increase or a decrease of the release probability. In connections with strong facilitation or strong depression (see Costa et al., 2013, Table 1), the magnitude of LTP or LTD of steady-state responses at ~4 Hz will then be ~15% less than LTP or LTD measured with single-pulses (calculated according to Equations (5)–(7) in Costa et al., 2013). For connections with less pronounced facilitation and depression (depression, facilitation and facilitation-depression in Table 1 in Costa et al., 2013), and lower presynaptic firing rates, the effect will be weaker, between 1 and 7%.

Further mechanisms limiting the level of postsynaptic activity include negative feedback on transmitter release via fast retrograde signaling (e.g., Pitler and Alger, 1992; Wilson and Nicoll, 2001; Freund et al., 2003; Hashimotodani et al., 2007), or activity-dependent changes of the extracellular level of adenosine (Pascual et al., 2005; Wall and Dale, 2008; Halassa et al., 2009; Lovatt et al., 2012), which has suppressive effect on synaptic transmission in the neocortex and hippocampus (e.g., Dunwiddie and Haas, 1985; Scanziani et al., 1992; Thompson et al., 1992; Kerr et al., 2013; Bannon et al., 2014; Zhang et al., 2015).

Finally, tight control of activity in neurons and neuronal networks is achieved by inhibition, including recurrent inhibition. Strong recurrent inhibition by design limits changes of activity level even during dramatic changes of external input allowing neuronal networks to operate in a regime of dynamically balanced excitation and inhibition (Wehr and Zador, 2003; Okun and Lampl, 2008; Ozeki et al., 2009; Dorn et al., 2010; Sun et al., 2010). Inhibitory plasticity can adjust the strength of inhibition and maintain the excitatory/inhibitory balance in neurons and neuronal networks (Vogels et al., 2011; Luz and Shamir, 2012).

These mechanisms, together with heterosynaptic plasticity, might contribute to a multi-level system of homeostatic control of synaptic changes and neuronal activity.

## Summary and conclusions

A long history of research supports the hypothesis that homosynaptic plasticity provides a powerful cellular mechanism of learning in a variety of biological systems. In this review we argued that a complimentary form of plasticity—heterosynaptic plasticity—represents a necessary cellular component for homeostatic regulation of synaptic weights and neuronal activity. The necessary properties of a homeostatic mechanism which acutely constrains the runaway dynamics imposed by Hebbian associative plasticity have been well-articulated by theoretical and modeling studies. The experimentally observed properties of heterosynaptic plasticity have introduced it as a strong candidate to fulfill this homeostatic role, and subsequent modeling studies which incorporate heterosynaptic plasticity into model neurons with Hebbian-type learning synapses have confirmed its ability to robustly provide stability and competition. In contrast, properties of homeostatic synaptic scaling, which is triggered by extreme and long lasting (hours and days) changes of neuronal activity, do not fit two crucial requirements for a hypothetical homeostatic mechanism needed to provide stability of operation in the face of on-going associative synaptic changes. Both the trigger and the time scale of homeostatic synaptic scaling are fundamentally different from those of the Hebbian-type plasticity.

Heterosynaptic plasticity, which operates on the same time scale and is triggered by similar activity episodes as homosynaptic plasticity, introduces a normalizing driving force that counterbalances a tendency for runaway dynamics of synaptic weights imposed by homosynaptic plasticity. As a result the system maintains synapses within an operational range, preserving the dynamic range for their changes. This allows it to modify synapses in response to a new experience—new learning. Segregation of synaptic weights and competition between synapses are achieved by the differential driving forces for the weight changes at active (homosynaptic) and inactive (heterosynaptic) synapses. At strongly activated homosynaptic sites, the associative driving force may be dominant, leading to net potentiation or depression of these sub-populations of synapses. Concurrently, the stabilizing effect of heterosynaptic plasticity dominates at the vast number of synapses which are inactive at that moment. As a consequence, every spike, or burst of spikes, becomes a homeostatic signal to the cell. Because homosynaptic and heterosynaptic changes are induced by the same activity patterns, and take place on the same time scale, the weight of a synapse is determined by the balance of homosynaptic LTP, homosynaptic LTD, and the normalizing force of heterosynaptic plasticity. This allows networks to update the relative strength of inputs while keeping synapses within their operational range, preserving their abilities for further adjustments, and maintaining the activity of neurons and networks in a stationary regime. Importantly, heterosynaptic plasticity allows robust homeostasis of synaptic weights and activity over a wide range of parameters: details of STDP rules, Ca^2+^ thresholds, and frequencies and correlations of presynaptic activity. Therefore, heterosynaptic plasticity expresses all of the desired features of an intrinsic homeostatic mechanism for stabilizing synaptic weight dynamics after learning.

A state of the neural system, e.g., controlled by different neuromodulators, may influence the relative balance of homo- and hetero-synaptic plasticity promoting either associative changes or synaptic homeostasis. Thus, hetero- and homo-synaptic forms of plasticity interact, with their balance depending on the state of the network, and therefore have to be studied in combination as integrative components of the whole plasticity system.

### Conflict of interest statement

The authors declare that the research was conducted in the absence of any commercial or financial relationships that could be construed as a potential conflict of interest.
